# Congenital adrenal hyperplasia in patients with adrenal tumors: a population-based case–control study

**DOI:** 10.1007/s40618-022-01933-0

**Published:** 2022-10-21

**Authors:** F. Sahlander, J. Patrova, B. Mannheimer, J. D. Lindh, H. Falhammar

**Affiliations:** 1 Department of Molecular Medicine and Surgery, Karolinska Institutet, Stockholm, Sweden; 2 Department of Medicine, Falu Hospital, 791 82 Falun, Sweden; 3 Center for Clinical Research Region Dalarna, Falun, Sweden; 4 Department of Clinical Science and Education at Södersjukhuset, Karolinska Institutet, Stockholm, Sweden; 5 Department of Laboratory Medicine, Division of Clinical Pharmacology, Karolinska University Hospital Huddinge, Karolinska Institutet, Stockholm, Sweden; 6 Department of Endocrinology, Karolinska University Hospital, Stockholm, Sweden

**Keywords:** 21-Hydroxylase deficiency, Adrenal incidentaloma, Adrenocortical cancer, Adrenalectomy, Adrenal crisis

## Abstract

**Purpose:**

Congenital adrenal hyperplasia (CAH) has been associated with adrenal tumors (ATs) but the relationship is still unclear. The aim was to investigate if CAH was more common in patients with adrenal tumors and their characteristics.

**Methods:**

Using national registers all patients with an AT diagnosis (cases) and selected matched controls without AT diagnosis were included from 1st January 2005 to 31st December 2019. The patients with a CAH diagnosis were scrutinized in detail.

**Results:**

ATs were diagnosed in 26,573 individuals and in none of 144,124 controls. In 20 patients with ATs and 1 control, a CAH diagnosis was present. The odds for having CAH in patients with ATs was 109 (95% CI 15–809; *P* < 0.0001). Among cases, 5 had a CAH diagnosis before the discovery of ATs and 15 afterwards. Half were females and two had been screened for CAH neonatally. The mean age when the ATs was discovered was 55.6 years. Adrenalectomy was performed in seven patients. Five patients had unilateral adrenalectomy before the CAH diagnosis and did not have any glucocorticoid protection. After the CAH diagnosis, 15 were initiated on glucocorticoids and 6 on mineralocorticoids. The majority diagnosed with CAH before index date had classic CAH. In individual diagnosed after index date, only three had classic CAH. The rest had nonclassical CAH. During the follow-up time of 9 years, six deceased, two of them in an adrenal crisis.

**Conclusions:**

The prevalence of CAH was greater in patients with ATs than in patients without. In all patients with ATs, CAH should be considered.

## Introduction

Congenital adrenal hyperplasia is a group of autosomal recessive disorders affecting different enzymes required for the cortisol synthesis in the adrenal glands [1]. The most common variant, 21-hydroxylase deficiency (21OHD), accounts for up to 99% of CAH cases [1, 2]. The deficient enzyme, 21-hydroxylase, causes an impaired synthesis of cortisol and aldosterone. By negative feedback, the ACTH levels increase and the steroid precursors are shunted into adrenal androgen production [3]. Three phenotypes can be seen, salt-wasting (SW) with severe cortisol and aldosterone deficiency as well as androgen excess, simple virilizing (SV) with cortisol deficiency and androgen excess but with milder aldosterone deficiency and non-classic (NC) CAH with typically only mild androgen excess [1]. SW and SV CAH often present with ambiguous external genitals in girls and are collectively known as classic CAH. Infants with SW CAH will die in a salt-wasting adrenal crisis within a few weeks of life if not treated, while individuals with SV CAH survive undiagnosed if not diagnosed due to ambiguous genitals in females or precocious puberty in both sexes [1, 4, 5]. Cases of SV CAH may not be diagnosed until adulthood, especially in low- to middle-income countries [6]. To save lives, neonatal screening has been introduced in many countries and in those countries all cases of classic CAH are diagnosed within the first week after birth while NC cases often are missed [7, 8].

Adrenal tumors are common, especially adrenal incidentalomas, i.e., tumors found accidentally, and adrenal incidentalomas are encountered in 1.9% of all individuals having a computed tomography (CT) where the adrenals can be visualized and in 3% of autopsies [9]. Undiagnosed CAH can be a cause of adrenal tumors [10, 11], even in very old age and can initially be suspected of being an adrenocortical cancer (ACC) due to their size and radiological characteristics [12]. International guidelines do not recommend general screening for CAH in patients with adrenal incidentalomas [13], however, a meta-analysis showed that 0.8–5.9% of all adrenal incidentalomas had CAH [14]. In patients with diagnosed CAH, adrenal masses including tumors are common [15, 16]. In a recent meta-analysis 29.3% of individuals with CAH examined with imaging had an adrenal tumor [17], a quarter of these were myelolipomas. Total adrenal volume and hyperplasia were associated with poor control and long-term outcomes [15, 16, 18]. ACTH is considered the driver for adrenal hyperplasia and tumor formation [17], especially in myelolipomas [19].

The aims of this study were to investigate if there was an increased prevalence of CAH in patients with adrenal tumors, and if the adrenal tumor preceded the CAH diagnosis or not. Moreover, we wanted to see to what extent adrenalectomy had been performed and what the long-term outcomes were.

## Methods

This was a retrospective, register-based study encompassing the entire Swedish population. By means of the Swedish personal identity number linkage between several national registers was possible. All cases of specialist outpatient care or hospitalization with a first-ever ICD10 code of D44.1 (Neoplasm of uncertain behavior of adrenal gland) and/or D35.0 (benign neoplasm of adrenal gland) from 1st January 2005 to 31st December 2019 were identified. Controls, matched by sex, age and place of residence, without an adrenal tumor diagnosis where then randomly selected by Statistic Sweden in a ratio of four controls to one case. All cases and controls with an ICD10 code of E25.0 (Congenital adrenogenital disorders associated with enzyme deficiency) were identified and the registers were reviewed manually to verify that the diagnosis was correct. E.g., to be classified as having CAH, the patient had to receive the diagnosis code E25.0 and/or E25.9 at least three times and been seen regularly at a Department of Endocrinology or Internal Medicine. Many secondary hospitals did not have a Department of Endocrinology, so the local endocrinologist was probably registered under their Department of Internal Medicine instead. Also, the ICD10 codes preceding the CAH code were taken into account to see if they made the diagnosis CAH plausible. E.g., if a young adult female was initially diagnosed with oligomenorrhea, then hirsutism, then polycystic ovarian syndrome and then at last with CAH for at least three times the CAH diagnosis was considered correct. Patients with suspected SW CAH where the exact age of diagnosis was not available, the age was set at 0 years, in line with previous studies [4, 5]. All born 1986 or later was assumed to have been screened for CAH since 1986 was the year of the introduction of the neonatal CAH screening in Sweden [20]. The registers used were the National Patient Register (containing all inpatient or/and specialist outpatient care) (data retrieved from 1997 to 2019), the Cause of Death Register (data retrieved from 2005 until 2020) and The Swedish Prescribed Drug Register (data retrieved from mid-2005 to 2019). All these registers are held by the National Board of Health and Welfare. All data were de-identified before delivery.

The study was approved by the Ethical Review Board in Sweden, and formal consent was waived due to the study's retrospective epidemiological design.

### Statistical analysis

Mean ± SD was used for continuous variables since all data were normally distributed. Comparisons were performed using Student’s *t* test for continuous and Fisher’s exact test or odds ratio (OR) calculations with 95% confidence interval (CI) for categorical variables. A *P* value < 0.05 was considered statistically significant. SigmaStat 3.0 for Windows (Systat Software Inc., San Jose, California) was used for all calculations.

## Results

An adrenal tumor was diagnosed in 26,573 individuals and 144,124 matched controls without an adrenal tumor was identified. Of these 276 patients with adrenal tumor and 1748 controls were born 1986 or later, i.e., around 1% had been screened neonatally for CAH. Of all patients with an adrenal tumor, 38 had at least one diagnosis of CAH. Among the controls, the corresponding number was 3. However, after review of all the ICD10 codes from in- and outpatient care, what department had deployed what ICD10 codes, prescribed medications, cause of death register and cancer register, only 20 patients with adrenal tumor and 1 control remained. Thus, 20/26573 (0.75‰) of those with adrenal tumor had CAH compared to 1/144124 (0.0007‰) controls (OR 109, 95% CI 15–809; *P* < 0.0001).

Detailed information on the 20 individuals with CAH and adrenal tumor can be seen in Table 1. Of cases, 5 (25%) had received a CAH diagnosis before the discovery of an adrenal tumor and 15 (75%) afterwards. Half of the total cohort of patients with adrenal tumor and CAH were females. Only two (10%) had been screened for CAH neonatally of which one was a suspected classic CAH probably found in the neonatal period and the other a NC which was probably missed in the neonatal screening. The mean age when the adrenal tumor was discovered was 55.6 years. The majority received follow-up at five Departments of Endocrinology in tertiary (university) hospitals, of which the Karolinska University Hospital had seen eight patients with adrenal tumor and CAH, the other tertiary hospitals four and the rest at eight different Departments of Endocrinology or Internal Medicine at secondary hospitals. Around a third (7 out of 20) had had an adrenalectomy. In patients with CAH before the diagnosis of adrenal tumor (five patients), two had bilateral adrenalectomy, one of these had a miscarriage and later, the procedure indicating that the adrenalectomy was done to improve fertility. In all patients with adrenal tumor that were diagnosed with CAH after the adrenal tumor was discovered, a third (five patients) had unilateral adrenalectomy before the CAH diagnosis and thus did not have any glucocorticoid protection. All adrenalectomies seemed to have been successful. One male patient in his 50 s and another in his 60 s were initially diagnosed with an adrenal tumor, then suspicion of ACC followed by adrenalectomy and then the final diagnosis CAH with a benign adrenal tumor. Once the CAH diagnosis was made, both glucocorticoid and mineralocorticoid substitution were initiated in one and in the other only glucocorticoid replacement, but the patient had a specific diagnosis of SV CAH, thus SV phenotype was suspected in both. In the total cohort, only 25% did not use glucocorticoid replacement after the CAH diagnosis, all suspected of having NC CAH. Mineralocorticoid replacement was used by a third, all presumed to be classic CAH. The mean follow-up time was slightly more than 9 years and 30% died, a third of them in an AC (2 and 9 years, respectively, after the adrenal tumor diagnosis).

**Table 1:** Patients with adrenal tumor and congenital adrenal hyperplasia (CAH), also subdivided into patients where CAH was found before or after the adrenal tumor

	Patients with CAH and adrenal tumor *n* = 20	CAH was found before *n* = 5	CAH was found after *n* = 15	*P* value
Female sex (*n*)	10 (50%)	2 (40%)	8 (53%)	1
Neonatal screened for CAH (*n*)	2 (10%)	1 (20%)	1 (7%)	0.45
Born overseas (*n*)	1 (5%)	0 (0%)	1 (7%)	1
Age when diagnosed with adrenal tumor (years)	55.6 ± 14.7	47.6 ± 15.7	58.2 ± 14	0.17
Age when CAH diagnosed (years)	48.5 ± 27.4	11.8 ± 26.4	60.7 ± 13.5	< 0.001
UH dealing with CAH and the adrenal tumor (*n*)	12 (60%)	4 (80%)	8 (53%)	0.60
Adrenalectomy (*n*)	7 (35%)	2 (40%)	5 (33%)	1
Unilateral (*n*)	5 (25%)	0 (0%)	5 (33%)	0.27
Bilateral (*n*)	2 (10%)	2 (40%)^&^	0 (0%)	0.053
Non-secreting adrenal tumor	20 (100%)	5 (100%)	15 (100%)	1
Benign adenoma (*n*)	20 (100%)	5 (100%)^&^	15 (100%)	1
Adrenocortical cancer or adrenal metastasis	0 (0%)^a^	0 (0%)	0 (0%)^a^	1
Use of glucocorticoids after CAH diagnosed (*n*)	15 (75%)	5 (100%)	10 (67%)	0.44
Use of mineralocorticoids after CAH diagnosed (*n*)	6 (30%)	4 (80%)	2 (13%)	0.014
Suspected classic CAH (*n*) or NC CAH (*n*)	7 (35%)	4 (80%)	3 (20%)^b^	0.014
	13 (65%)	1 (20%)	12 (80%)	
Follow-up time after adrenal tumor diagnosis (years)	9.2 ± 4.9	7.4 ± 4.5	9.8 ± 5	0.362
Alive at the end of follow-up (*n*)	14 (70%)	3 (60%)	11 (73%)	0.61
Died during follow-up and cause of death	AC *n* = 2 (33%)^c^	AMI *n* = 1 (50%)	AC *n* = 2 (50%)^c^	N/A
	AMI *n* = 1(17%)	Lung ca *n* = 1 (50%)	Suicide *n* = 1(25%)	
	Lung ca *n* = 1(17%)			
			T-accident *n* = 1 (25%)	
	Suicide *n* = 1(17%)			
	T-accident *n* = 1(17%)			

Continuous parameters are expressed as mean ± SD

Neonatal screening of CAH was initiated in 1986 in Sweden and all born after this date in Sweden was assumed to have been screened

*UH* university hospital, *ca* cancer, *AMI* acute myocardial infarction, *AC* adrenal crisis, *T-accident* traffic accident

^&^Two patients had both radiological and histopathological clear findings of large myelolipomas

^a^Two cases were initially suspected to be adrenocortical cancer, however, after adrenalectomy the diagnosis was changed to benign adenoma. It should be noted that those who did not have adrenalectomy the diagnosis of benign adenoma was probably made radiologically

^b^One was only on glucocorticoid replacement but had a specific SV CAH diagnosis

^c^One had previously had adrenalectomy without glucocorticoid replacement but was later initiated on glucocorticoids

The control with CAH was a female born before neonatal screening. She was initially diagnosed with hirsutism but in her late 20 s, she was diagnosed with CAH, probably NC CAH. She then continued her care at the Department of Endocrinology at a university hospital but did not receive glucocorticoid replacement.

When comparing those diagnosed with CAH before the adrenal tumor with those diagnosed after, those diagnosed with CAH before were probably mostly diagnosed during childhood (only one was diagnosed in the 50 s with probable NC), while those diagnosed with CAH after the adrenal tumor was around 60 years of age (Table 1). One woman, however, was first diagnosed with amenorrhea and hirsutism during adolescence, in her 20 s diagnosed with an adrenal tumor and a few months later with CAH, probably NC CAH. Mineralocorticoids were used more in those diagnosed with CAH earlier and 80% of them was suspected to have either SW or SV CAH, while in those diagnosed with CAH after the adrenal tumor, only 20% was suspected to have SV CAH and the rest NC CAH.

## Discussion

This is the first nationwide and largest study of CAH in patients with adrenal tumors. We found that the prevalence of CAH was much higher in patients with adrenal tumors than in controls without any diagnosed adrenal tumor. In the majority of patients, the CAH diagnosis was established after the adrenal tumor was found. Most of these probably had NC CAH but a few had SV CAH, and these had survived in spite of not being on a glucocorticoid replacement, even during adrenalectomy. In total, 75% had been initiated on glucocorticoid replacement after the CAH diagnosis. During the almost 10-year follow-up period, the mortality rate was 30%, a third deceased due to an adrenal crisis.

The prevalence of CAH was hugely elevated in those with an adrenal tumor in the current study which is in line with previous studies showing that undiagnosed CAH affects some patients with an adrenal incidentaloma [11, 14]. ACTH is thought to be the driver of adrenal tumor development [17, 21]. ACTH levels is compensatory high in individuals with untreated CAH due to negative feedback from the low cortisol production [1]. Once replaced with glucocorticoids the ACTH levels normalize in many, but not all, patients with CAH [4, 18]. High ACTH levels act as a growth factor on adrenal cells and thus result in adrenal hyperplasia [17, 18]. If elevated ACTH levels also cause tumor formation and growth are not as clear but could be one of the reasons for the high prevalence of adrenal myelolipomas and adenomas in CAH [17]. Pituitary extracts injected in the adrenal glands was found to generate a myeloid phenotype indicating that ACTH may help in at least the development and growth of myelolipomas [22]. Patients with CAH that have been diagnosed with neonatal screening, thus receiving glucocorticoid replacement early, and then hopefully have good adherence to therapy most likely have a low risk of developing adrenal tumors.

An adrenal crisis is a lethal condition in patients with cortisol insufficiency and may be induced by stressful situations if not appropriate glucocorticoid coverage have been installed [23, 24]. Thus, an adrenalectomy in a patient with CAH without glucocorticoid protection can potentially pose a risk of developing a life-threatening adrenal crisis, especially with adjacent surgical complications. The symptoms of adrenal crisis may be diffuse and difficult to interpret in all ages [23, 25]. Interestingly, five patients with undiagnosed CAH (of which two probably had two SV CAH), had had a successful unilateral adrenalectomy in spite of not being on glucocorticoid replacement. However, some glucocorticoid activity has been demonstrated by adrenal steroid precursors in vitro [26]. Thus, this may have been enough to make these patients with classic CAH survive so long, even the adrenalectomy without being diagnosed with CAH. In NC CAH, though, only a third has partial cortisol insufficiency [27, 28], so patients with NC CAH usually manage severe stress without glucocorticoid replacement. Furthermore, glucocorticoids are often given just prior to surgery to prevent nausea [29], and may in fact have helped to save these cases with SV CAH and adrenalectomy from death. It should be noted that once glucocorticoid replacement has been installed the adrenal production of cortisol and steroid precursors are inhibited so stress doses of glucocorticoids are needed during infections and surgery which explains why patients with NC CAH may die of adrenal crisis [30]. This may be the reason two deaths happened of adrenal crisis years after the glucocorticoid replacement was initiated. Mortality has been shown to be increased in CAH and the main cause to be adrenal crisis [30].

In the work-up of an adrenal incidentaloma, it is important to exclude a malignant lesion, such as ACC or metastasis, and a hormone-producing tumor [9, 13]. Occasionally the adrenal tumor in a patient with an undiagnosed CAH can give question of an ACC due to suspicious radiological features [12]. Even though ACC is rarely found in patients with CAH [14, 31], ACC should still be considered in patients with CAH and concomitant adrenal tumor. Detailed steroid profile assessment with mass spectrometry-based could be considered as it can distinguish an ACC from a benign adrenal tumor [32]. Hormone-producing adrenal tumor in patients with CAH is also extremely rare with only two cases of pheochromocytoma found in patients with CAH [16, 33]. In the current study, we found a few cases with initial suspicion of ACC but was later changed to a benign adrenal tumor once the CAH had been diagnosed. We found no case of hormone-producing adrenal tumor in the patients with CAH. However, if an adrenal tumor is found in any patient, including patients with CAH, exclusion of malignancy and hormone-production from the adrenal tumor should be done.

The question if CAH should be excluded in patients with adrenal incidentaloma is unclear and it is not generally recommended in international guidelines which only advise on consideration of 17-hydroxyprogesterone (17OHP) measurements in bilateral tumors and when clinical suspicious arises [13]. Basal 17OHP levels are clearly elevated in classic CAH but basal 17OHP levels not so in NC CAH where ACTH stimulated 17OHP levels may be needed to reach the diagnostic level of 30 nmol/l or more [8]. There is a relationship with the size of the adrenal tumor(s) and 17OHP levels [10, 14]. Moreover, 17OHP levels may be increased by malignant tumors and bilateral macronodular adrenal hyperplasia so the results have to be interpreted with caution [13]. Thus, if a high 17OHP level is found *CYP21A2* mutation analysis should be performed, especially in levels between 30 and 60 nmol/L [14]. Since only 0.8–5.9% of adrenal incidentalomas are caused by undiagnosed CAH and bilateral adrenal tumors do not reliably predict CAH, only signs of hyperandrogenism in a patient with adrenal tumor should lead to investigation for CAH [14]. Maybe also patients from ethnicities with a high frequency of CAH (e.g. Ashkenazi Jews, Hispanics, Greeks) screening could be justified as well [34], even though the current study do not support this. One University Hospital had seen almost half of the patients with CAH and adrenal tumors which suggest that if you find a few you start looking for more. This together with the data showing that CAH is quite common in adrenal incidentalomas if you start investigating for it, while in the present study, it was very rare suggest that the knowledge about CAH is limited among physicians as well as surgeons and may result in unnecessary suffering. Large unclear adrenal tumors should be discussed in Multi-Disciplinary Team meetings before adrenalectomy and CAH should be considered.

Like all studies, the present one has not only limitations but also strengths. The study was register-based and misclassifications may have happened. Neither 17OHP levels nor genetic analysis results were available in the registers so we could not biochemically nor genetically confirm these CAH cases. On the other hand, we know from previous studies that more than 80% of patients with CAH in Sweden have their diagnosis confirmed by *CYP21A2* gene analysis [2]. Moreover, all register data were reviewed in detail to make sure the CAH diagnosis was correct. Only the most specific diagnosis of CAH, E25.0, was considered but E25.8 (Other adrenogenital disorders) and E25.9 (Adrenogenital disorder, unspecified) are sometimes used as well by physicians not well-versed in CAH care. There may be missed cases of CAH if the ICD code was not registered or if the diagnosis was registered late during the observation time of the study leading to less than three registrations and then not meeting inclusion criteria of this study. However, we assess that any missed cases do not significantly affect the relationship between the comparison groups. Additional cases of CAH in the group with adrenal tumors had only reinforced the significant difference between the groups. A sign that this review may have been too strict was that only 1 individual with CAH was found in the 144,124 controls and the prevalence of CAH in Sweden is 1:11,200 newborn [20]. Though, neonatal screening started in 1986 in Sweden and many patients with CAH were missed previously [2]. Furthermore, most adrenal tumors were found in older age so the controls were also mostly elderly where CAH is rare [35]. Since controls could not have been diagnosed with an adrenal tumor, this may also have decreased the number of CAH cases as they had not been exposed to specialized endocrine care to the same degree. If the medical investigation of the adrenal tumors in cases increased the likelihood of discovering a hitherto undiagnosed CAH, this could have inflated the risk estimate in the cases. The fact that 75% of all CAH diagnoses in the cases were made after the discovery of the adrenal tumor indicate that this may indeed be the case and that some of the observed association between CAH and adrenal tumor could be due to detection bias. Another limitation was that we did not have the medical files available to validate the data since a prerequisite to get access to these data were that the identity of the patients was unknown to us. Thus, we could not acquire, e.g., radiological data to be able to identify myelolipomas. Even though this was the largest study of its kind the number of patients with CAH was still limited. The strengths are that this is the first nationwide study and also the largest study in the field minimizing the risk of selection bias and the precision of the association estimates have improved substantially. Moreover, the follow-up time was almost a decade with 100% follow-up data which is quite unique compared to other studies.

## Conclusion

The prevalence of CAH was greater in patients with adrenal tumors than in patients without. The CAH diagnosis was mainly done after the adrenal tumor was found, sometimes even after an adrenalectomy. This exposes the patients with undiagnosed CAH for great danger of a lethal adrenal crisis. Centers that had previously diagnosed CAH in patients with adrenal tumors were more prone to find additional cases. In patients with a previous diagnosis of CAH, the finding of an adrenal tumor may be in the course of investigating why a patient had poor hormonal control and often led to bilateral adrenalectomy. In all patients with an adrenal tumor, CAH should be considered.
